# Expression of fatty acid‐binding protein‐4 in gastrointestinal stromal tumors and its significance for prognosis

**DOI:** 10.1002/jcla.24017

**Published:** 2021-09-24

**Authors:** Wei‐Jie Zang, Zi‐Niu Wang, Yi‐Lin Hu, Hua Huang, Peng Ma

**Affiliations:** ^1^ Department of Gastrointestinal Surgery Affiliated Hospital of Nantong University Nantong China; ^2^ Department of Clinical Biobank Nantong University Affiliated Hospital of Nantong University Nantong China; ^3^ Medical School of Nantong University Nan Tong China; ^4^ Department of Pathology Affiliated Hospital of Nantong University Nantong China

**Keywords:** FABP4, GISTs, malignant, phenotype, prognosis

## Abstract

**Background:**

Fatty acid‐binding proteins (FABPs) have been found to be involved in tumorigenesis and development. However, the role of FABP4, a member of the FABPs, in GISTs (Gastrointestinal stromal tumors) remains unclear. This study aimed to investigate the expression of FABP4 and its prognostic value in GISTs.

**Methods:**

FABP4 expression in 125 patients with GISTs was evaluated by immunohistochemical analysis of tissue microarrays. The relationship between FABP4 expression and clinicopathological features and prognosis of GISTs was analyzed.

**Results:**

Multiple logistic regression analysis showed that expression of FABP4 correlated with tumor size and mitotic index. Furthermore, FABP4 level, tumor size, mitotic index, and high AFIP‐Miettinen risk were independent prognostic factors in GISTs. The Kaplan‐Meier survival curve showed that the 5‐year survival rate of patients with high‐FABP4 expression GISTs was lower.

**Conclusions:**

These results suggested that high‐FABP4 expression might be a marker of malignant phenotype of GISTs and poor prognosis.

## Graphical abstract

This study found that the high expression of FABP4 was associated with poor prognosis in patients with GISTs, suggesting that FABP4 could be used as an independent indicator to judge the prognosis of GISTs and serve as a potential therapeutic target.

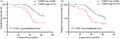

## 1 INTRODUCTION

Gastrointestinal stromal tumor (GIST) is defined as a stromal tumor of spindle or epithelioid cells that is primary in the gastrointestinal tract, greater omentum, and mesentery with a KIT (CD117 stem cell factor receptor) positive stain. It is a distinct tumor from the typical smooth muscle and neurogenic tumor, and it is a separate disease but it can differentiate into either smooth muscle or nerve. GISTs are the most common mesenchyme‐derived tumors of the gastrointestinal tract. They are different from interstitial cells of Cajal.^1^, ^2^ GISTs are characterized by the biological characteristics of benign or malignant tumors. Complete radical resection is the most effective treatment for localized GISTs and potentially resectable GISTs.^3^ However, some patients have no chance of surgery due to the late stage of the tumor or special lesions such as the low rectal GISTs that require combined anal resection.^4^ The emergence of specific molecular targeted drugs such as imatinib changes the treatment mode of GISTs and significantly improves the patient's treatment effect.^5^ For resistant patients, only sunitinib, regorafenib, and other second‐line and third‐line molecular targeting drugs can be used to control the development of tumor, but the effect is poor.^6^ Therefore, the discovery of new specific biomarkers with clinical and prognostic significance is very important for treating GISTs.

Fatty acid‐binding proteins (FABPs) are a member of the intracellular lipid binding protein superfamily, which mainly exists in the cytoplasm of vertebrates and invertebrates. FABPs are small‐molecule proteins widely expressed in mammalian tissues and cells.^7^, ^8^, ^9^, ^10^, ^11^, ^12^, ^13^ They are highly conserved in evolution and exhibit certain tissue specificity. So far, nine FABPs have been identified, named FABP1‐9, which are liver type, intestinal type, heart type, fat type, skin type, ileal type, brain type, peripheral nerve type, and testicular type.^14^ Previous studies have shown that FABPs are intracellular fat partners whose main function is to participate in fatty acid absorption, accumulation, and intracellular transport. This includes transporting fatty acids to fat particles, endoplasmic reticulum, mitochondria, lysosomes, and nucleus for storage or oxidative metabolism; participating in signal transmission and cell membrane synthesis; regulating enzyme activity or gene transcription; and even serving as an autocrine or paracrine signal transporter out of the cell, thereby participating in the regulation of cell proliferation, differentiation, apoptosis, and other functions.^15^, ^16^ These studies also demonstrate the enormous potential of FABPs as the indicators of disease diagnosis and prognosis.

FABP4 is the most characteristic and most studied protein belonging to the FABPs family.^17^ It is expressed in the cytoplasm and is lipophilic. It consists of 134 amino acids and is secreted by fat cells and macrophages.^18^, ^19^ In recent years, FABP4 has gained a lot of interest, especially in terms of cell differentiation, glycolipid metabolism, and inflammatory response. It is a key factor in linking metabolic diseases and insulin resistance, lipid metabolism disorders, and atherosclerosis.^20^, ^21^, ^22^, ^23^, ^24^, ^25^ Also, it is associated with a variety of tumorigenesis developments.^26^ However, whether FABP4 plays a role in the occurrence and development of GIST has not been studied. Therefore, the aim of our study was to detect the expression of FABP4 protein in GIST tumor samples, analyze the relationship between FABP4 expression level and clinicopathological features of patients with GISTs, and further analyzed the effect of FABP4 protein expression on the prognosis of GIST.

## 2 MATERIALS AND METHOD

### 2.1 Research sample

The study included 125 patients with GISTs who were treated at the Nanjing First Affiliated Hospital of Nanjing Medical University and Affiliated Hospital of Nantong University from 2003 to 2010. The inclusion criteria met the requirements as follows: (1) patients underwent R0 radical resection, and tumor has no distant metastasis. (2) They were diagnosed with GISTs by histopathological features and positive for CD117 immunohistochemical (IHC) analyses. (3) No targeted therapy or chemotherapy or radiotherapy was performed before surgery. (4) The general clinical and pathological data are complete. Exclusion criteria were as follows: (1) Patients who have received radiotherapy and chemotherapy. (2) The clinical data are incomplete, or routine immunohistochemical testing has not been performed. (3) Patients with previous history of other tumors. (4) GIST has been a distant transfer. They were not treated with tyrosine kinase inhibitors, including imatinib and sunitinib, before the surgery. Patients were examined for clinicopathological data such as sex, age, tumor size, number of lesions, primary tumor site, mitotic figures per 50 high‐power field (HPF), Miettinen risk stratification, 5‐year overall survival (OS), and disease‐free survival (DFS). OS and DFS refer to the interval from the time of surgery to death or recurrence, respectively. Postoperative follow‐up began in June 2003 and ended in June 2015 (median follow‐up time was 47 months; Range, 4–65 months). Postoperative follow‐up patients underwent an abdominal computed tomography (CT) examination every 6 months, and the patients suspected of gastrointestinal recurrence were examined by enhanced CT. Patients with suspected gastrointestinal recurrence were examined for gastroscopy or gastrointestinal angiography. Each of the patients involved in this study provided informed consent before sample collection, and the study protocol was approved by the Clinical Research Ethics Committee of the hospital.

### 2.2 Tissue microarray construction and IHC analysis

A tissue microarray (TMA) system (Quick‐Ray, UT06; UNITMA) was used from the Department of Clinical Pathology, at the Affiliated Hospital of Nantong University. TMA was performed following a previously described protocol.^27^ Based on the results of HE staining of pathological sections, the representative cancer nests were selected and labeled on the corresponding donor paraffin blocks. Then, TMA was analyzed by IHC, and the primary antibody was replaced with phosphate‐buffered saline (PBS) for negative control. The sections were dewaxed and rehydrated, in xylene and gradient ethanol, respectively. They were then incubated with primary antibody against FABP4 (1:100 dilution, Proteintech, 12802‐1‐AP) overnight at 4°C. Further, they were washed with PBS three times and incubated with the secondary antibody (horseradish peroxidase–conjugated anti‐mouse antibody) for 1 h at room temperature. Immunoreactivity was evaluated using a Vectastain Elite ABC kit (Vector Laboratories). The films were viewed in a double‐blind manner by two experienced pathologists, and the ratio and strength of FABP4‐positive cells were evaluated. From weak to strong, the dyeing strength was divided into four grades: 0, 1, 2, and 3. The ratio of positive cells was scored as follows: 0%–25% for 0 points, 26%–50% for 1 point, 51%–75% for 2 points, and 76%–100% for 3 points. The FABP4 immunohistochemistry score (IHS) was based on the ratio of positive cells multiplied by the strength score, with the IHS of 3 or less being defined as low expression and greater than or equal to 4 as high expression.^27^, ^28^, ^29^

### 2.3 Statistical analysis

SPSS 22.0 statistical software (SPSS) was used for data analysis. The clinical significance of FABP4 expression in GISTs and its various clinical parameters was analyzed by univariate and multivariate statistical analysis, using chi‐square test. Kaplan‐Meier was used to draw the survival curve, and log‐rank test was used to compare the survival rate. Independent risk factors were identified using the Cox model for univariate and multivariate analyses. A *p* value <0.05 indicated a statistically significant difference.

## 3 RESULT

### 3.1 Clinical features

A total of 125 patients with GISTs were enrolled in this study (64 male and 61 female; average age 57 years [16–96 years]): 63 were aged more than 60 years, and 62 were aged less than or equal to 60 years. Further, 35 patients had a tumor diameter of <5 cm, 58 patients had a tumor diameter of 5–10 cm, and 32 patients had a tumor diameter of ≥10 cm. The mitotic index of 59 patients (per 50 HPF, 0.375‐mm‐diameter field of view of the microscope) was 0–5/HPF, the mitotic index of 38 patients was 6–10/HPF, and the mitotic index of 28 patients was >10/HPF. The primary site of the tumor was the stomach in 58 patients, the intestine in 47 patients, and other organs in 20 patients. Also, 117 patients had a single lesion and 8 patients had multiple lesions. The AFIP‐Miettinen risk classification assessment revealed that 73 patients were at the low to medium risk level and 52 patients at the high‐risk level.

### 3.2 Expression level and location of FABP4 in GIST

**FIGURE 1:**
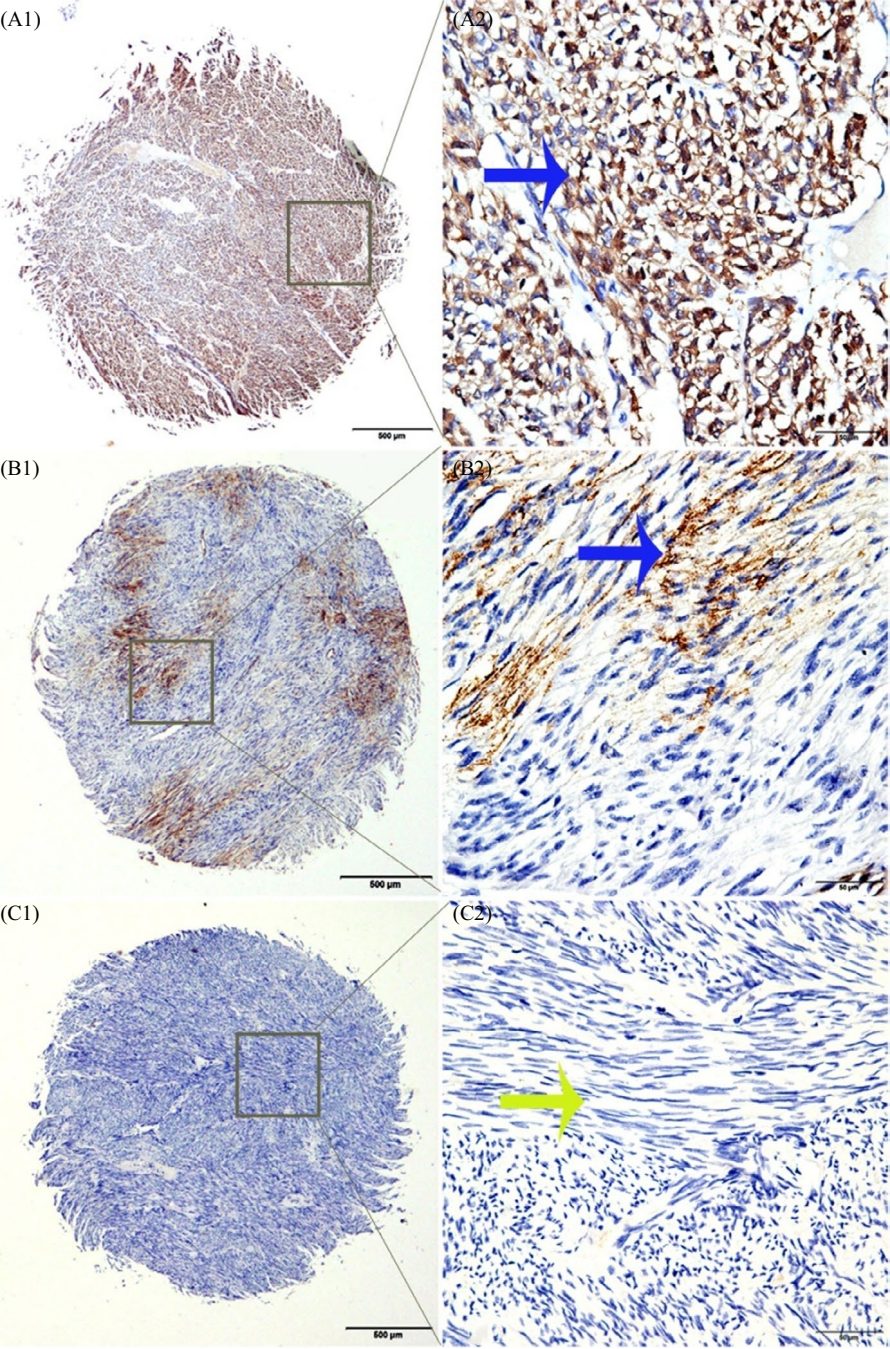
Immunohistochemical staining of FABP4 in clinical tissue samples of GISTs. A1: High cytoplasmic staining of FABP4 in the tissue microarray samples. A2: Specific high positive staining for FABP4 in the cytoplasm. B1: Low cytoplasmic staining of FABP4 in gastrointestinal stromal tumor tissues. B2: Specific low positive staining for FABP4 in the cytoplasm. C1 and C2: Negative staining for FABP4. Original magnification: A1, B1, C1 × 40; A2, B2, C2 × 400

In this study, immunohistochemical analysis was used to analyze the expression level and cell localization of FABP4 in GIST. FABP4 was mainly expressed in the cytoplasm of GIST cells. Of the 125 patients with GIST, 37 (29.60%) had high‐FABP4 expression and 88 (70.40%) specimens had low or no expression (Figure 1).

### 3.3 Correlation between FABP4 expression and clinicopathological components

This study further investigated the relationship between FABP4 expression and clinicopathological components in 125 patients. The data showed that FABP4 expression correlated with the increase in tumor size (*p* = 0.002), mitotic index (*p* < 0.001), and AFIP‐Miettinen risk stratification (*p* = 0.001). It is not related to other clinicopathological parameters such as sex, age, number of lesions, and tumor location (*p* > 0.05) (Table 1). Multiple logistic regression analysis showed that the expression of FABP4 was significantly associated with tumor size (odds ratio = 2.115, 95% confidence interval [CI] = 1.104−4.204, *p* = 0.024) and mitotic index (odds ratio = 2.623, 95% confidence interval [CI] = 1.408−4.889, *p* = 0.002).

**TABLE 1:** Association of FABP4 expression with clinical characteristics and selected biological markers of GIST

Groups	No.	Low or no expression (%)	High expression (%)	*p* Value
Total	125	88 (70.40)	37 (29.60)	
Gender				
Female	61	44 (72.13)	17 (27.87)	0.679
Male	64	44 (68.75)	20 (31.25)	
Age (years)				
≦60	62	43 (69.35)	19 (30.65)	0.800
>60	63	45 (71.43)	18 (28.57)	
Tumor size (cm)				
<5	35	30 (85.71)	5 (14.29)	0.002*
5–10	58	43 (74.14)	15 (25.86)	
≧10	32	15 (46.88)	17 (53.12)	
Mitotic index (per 50 HPFs)				
0–5	59	52 (88.14)	7 (11.86)	<0.001*
6–10	38	25 (65.79)	13 (34.21)	
>10	28	11 (39.29)	17 (60.71)	
Number of lesions				
Single nodule	117	83 (70.94)	34 (29.06)	0.613
Multiple nodules	8	5 (62.50)	3 (37.50)	
Tumor location				
Stomach	58	44 (75.86)	14 (24.14)	0.370
Intestine	47	32 (68.09)	15 (31.91)	
Others	20	12 (60.00)	8 (40.00)	
AFIP‐Miettinen risk				
Very low–Moderate risk	73	60 (82.19)	13 (17.81)	0.001*
High risk	52	28 (53.85)	24 (46.15)	

Note

*p < 0.05.

Abbreviations: FABP4, fatty acid‐binding protein 4; GIST, gastrointestinal stromal tumors; HPFs, high‐power fields.

### 3.4 Survival analysis

**FIGURE 2:**
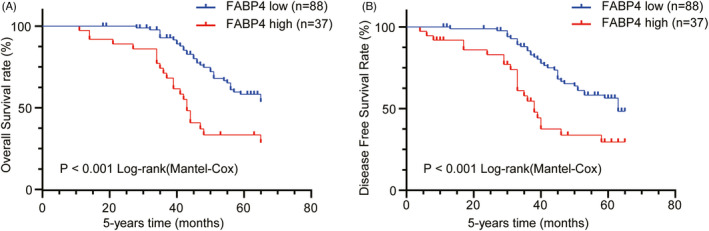
Kaplan‐Meier survival curves of patients with GIST. (A, B) Patients with high‐FABP4 expression (red line) had a poor overall survival (OS) and disease‐free survival (DFS) compared with patients with low and no FABP4 expression (blue line). OS and DFS curves were performed using GraphPad software (Prism Version 8, Inc.)

The Kaplan‐Meier survival curve showed that the 5‐year OS (*p* < 0.001) and DFS (*p* < 0.001) were significantly lower in patients with high‐FABP4 expression GIST compared with patients with low and no FABP4 expression (Figure 2A,B). And the median survival of the 125 patients was 47 months. The univariate analysis revealed that the prognosis of patients with GIST was related to FABP4 expression (*p* < 0.001), tumor size (*p* = 0.026), mitotic index (*p* = 0.027), and AFIP‐Miettinen risk levels (*p* < 0.001). The multivariate analysis suggested that poor prognosis in patients with GIST was significantly associated with high‐FABP4 expression (*p* = 0.021) and high AFIP‐Miettinen risk (*p* < 0.001) (Table 2).

**TABLE 2:** Univariate and multivariate analysis of prognostic factors in GIST for 5‐year overall survival rate

	Univariate analysis				Multivariate analysis			
	HR	*p* > |z|	95% CI		HR	*p* > |z|	95% CI	
FABP4 expression High vs. Low	2.611	<0.001*	1.523	4.476	2.052	0.021*	1.116	3.775
Age (years) ≤60 vs. >60	1.364	0.247	0.806	2.309				
Gender Male vs. Female	0.901	0.695	0.536	1.516				
Tumor size (cm) 5 vs. 5–10 vs. ≥10	1.487	0.026*	1.050	2.106				
Mitotic index (per 50 HPFs) 0–5 vs. 6–10 vs. >10	1.450	0.027*	1.044	2.015				
Number of lesions Single vs. Multiple	1.330	0.543	0.531	3.333				
Tumor location Stomach vs. intestine	1.051	0.788	0.732	1.508				
AFIP‐Miettinen risk Very low–Moderate risk vs. High risk	3.701	<0.001*	2.163	6.333	3.576	<0.001*	1.961	6.519

Note

*p < 0.05.

Abbreviations: CI, confidence interval; FABP4, fatty acid‐binding protein 4; GIST, gastrointestinal stromal tumors; HPFs, high‐power fields.

## 4 DISCUSSION

The main function of FABP4 is to control lipid metabolism; participate in inflammatory response, cell growth, and differentiation; and regulate apoptosis. FABP4 expression is regulated by insulin and insulin‐like growth factor 1 (IGF‐1), dexamethasone and fatty acids, peroxisome proliferator−activated receptor gamma (PPARγ), and PPARγ agonists.^30^ Lipids have an important regulatory function and serve as energy providers in organisms. Previous studies proved that FABP4 expression levels were inconsistent in different tumor tissues and played different roles. Hancke et al.^31^ found that the serum levels of FABP4 were relatively high in patients with breast cancer compared with healthy women, and their levels positively correlated with tumor size, stage, and lymph node metastasis. Nieman et al.^32^ found that the growth ability of metastatic tumors in FABP4‐expressing mice decreased in ovarian cancer. Mathis C et al.^33^ found that exogenous FABP4 increased the invasive ability of bladder cancer cells in vitro, which might be achieved by binding to fatty acids or the phosphatidylinositol 3‐kinase‐protein kinase pathway. These studies suggested that FABP4 played a role in promoting cancer in the development of tumors. However, studies by Ohlsson et al.^34^ found that the higher the degree of malignancy, the lower the expression level of FABP4 mRNA and protein in bladder tumor tissues. Studies on the prostate cancer cell line DU145 revealed that FABP4 was not expressed in cells; subsequent introduction of FABP4 into DU145 cells revealed that the proliferation was significantly inhibited.^35^ Therefore, whether FABP4 played a role in promoting or suppressing cancer growth is controversial.

Studies showed that lipid biosynthesis increased dramatically in highly invasive GISTs to meet the needs of rapidly proliferating tumor cells.^36^ Fatty acid synthase (FASN) is expressed in highly invasive GISTs, can be used as a predictor of survival alone, and may serve as a therapeutic target for GISTs.^37^ FASN and FABP4 play important roles in the synthesis and transport of long‐chain fatty acids, respectively. Hence, the role of FABP4 in GISTs needs further exploration.

TMA IHC was used in this study. Only 38 (30.4%) of 125 patients with GISTs were observed to have high‐FABP4 expression. However, FABP4 expression was found to be associated with clinicopathological features such as large tumor size, multiple lesions, and high AFIP‐Miettinen risk stratification. The results suggested that FABP4 could be used as a malignant phenotype of GIST.

The clinical studies focused on GIST tumor size, primary site, mitotic index, number of lesions, and, so forth, as indicators to determine the degree of GIST risk and prognosis. However, some patients with disease progression could not be judged very well using these indicators. For example, tumors measuring less than 2 cm had distant metastasis, and some patients with a high level of malignancy had long‐term survival. Therefore, finding molecules that affect the progress of GISTs is very important to judge the degree of GIST risk and prognosis. Studies found that the expression of miR‐148b‐3p in GIST increased with increasing risk, while the low expression of miR‐148b‐3p in high‐risk GIST suggested that recurrence and metastasis were more likely to occur.^38^ Studies suggested that the level of DKK4 was significantly higher in high‐risk patients with GIST than in low‐risk patients and could be used as a prognostic indicator.^39^ The present study found high‐FABP4 expression in some GIST tissues. Further, patients with increased FABP4 expression had poor prognosis. The 5‐year survival rate significantly reduced. Hence, FABP4 expression is a novel and valuable marker for judging the prognosis of GIST and is an independent indicator of the degree of risk. There are some limitations to this study. We lack larger samples and multi‐regional studies. In addition, we still lack the related cytology and RNA level of deeper studies. Further research is needed to explore the therapeutic value of FABP4 and to study the potential role of FABP4 in GIST.

## CONFLICT OF INTEREST

The authors report no proprietary or commercial interest in any product mentioned or concept discussed in this article.

## AUTHOR CONTRIBUTIONS

Peng Ma and Hua Huang led study design and prepared the study. Wei‐Jie Zang performed data analysis and interpretation. Zi‐Niu Wang provided data collection. Yi‐Lin Hu provided data collection. All authors read and approved the final study.

## DECLARATION OF FIGURES AUTHENTICITY

All figures submitted have been created by the authors who confirm that the images are original with no duplication and have not been previously published in whole or in part.

## Data Availability

The data that support the findings of this study are available on request from the corresponding author.
